# Genome-wide association analysis of feed intake and residual feed intake in Nellore cattle

**DOI:** 10.1186/1471-2156-15-21

**Published:** 2014-02-11

**Authors:** Miguel HA Santana, Yuri T Utsunomiya, Haroldo HR Neves, Rodrigo C Gomes, José F Garcia, Heidge Fukumasu, Saulo L Silva, Gerson A Oliveira Junior, Pâmela A Alexandre, Paulo R Leme, Ricardo A Brassaloti, Luiz L Coutinho, Thiago G Lopes, Flávio V Meirelles, Joanir P Eler, José BS Ferraz

**Affiliations:** 1 Faculdade de Zootecnia e Engenharia de Alimentos – USP, Av. Duque de Caxias Norte, 225, 13635-900 Pirassununga, São Paulo, Brazil; 2 Faculdade de Ciências Agrárias e Veterinárias, UNESP - Univ Estadual Paulista, Jaboticabal, 14884-900 São Paulo, Brazil; 3 Empresa Brasileira de Pesquisa Agropecuária, CNPGC/EMBRAPA, BR 262 km 4, 79002-970 Campo Grande, Matogrosso do Sul, Brazil; 4 Faculdade de Medicina Veterinária de Araçatuba, UNESP – Univ Estadual Paulista, Araçatuba, 16050-680 São Paulo, Brazil; 5 Escola Superior de Agricultura Luiz de Queiroz, USP – Univ. de São Paulo, 13418-900 Piracicaba, São Paulo, Brazil

## Abstract

**Background:**

Feed intake plays an important economic role in beef cattle, and is related with feed efficiency, weight gain and carcass traits. However, the phenotypes collected for dry matter intake and feed efficiency are scarce when compared with other measures such as weight gain and carcass traits. The use of genomic information can improve the power of inference of studies on these measures, identifying genomic regions that affect these phenotypes. This work performed the genome-wide association study (GWAS) for dry matter intake (DMI) and residual feed intake (RFI) of 720 Nellore cattle (*Bos taurus indicus*).

**Results:**

In general, no genomic region extremely associated with both phenotypic traits was observed, as expected for the variables that have their regulation controlled by many genes. Three SNPs surpassed the threshold for the Bonferroni multiple test for DMI and two SNPs for RFI. These markers are located on chromosomes 4, 8, 14 and 21 in regions near genes regulating appetite and ion transport and close to important QTL as previously reported to RFI and DMI, thus corroborating the literature that points these two processes as important in the physiological regulation of intake and feed efficiency.

**Conclusions:**

This study showed the first GWAS of DMI to identify genomic regions associated with feed intake and efficiency in Nellore cattle. Some genes and QTLs previously described for DMI and RFI, in other subspecies (*Bos taurus taurus*), that influences these phenotypes are confirmed in this study.

## Background

Feed intake plays an important economic role in cattle growth and may represent the greatest costs in beef cattle, both in beef cattle finishing systems and calves production [1]. Feed intake is evaluated by dry matter intake (DMI) in cattle, which is associated with weight gain, carcass traits and feed efficiency [2]. It is, therefore, a relevant variable for the entire meat production system and, possibly, a trait to be included in genetic breeding programs.

Although genetic breeding foci primarily on growth and reproductive traits, other variables must be taken into account because increased weight gain rates may lead to a concomitant increase in the adult size generating higher maintenance costs of animals [3]. Thus, the objectives of genetic breeding must be adequately delineated to attain balance between weight gain rates and other essential characteristics, such as reproduction [4], meat quality and feeding efficiency [3].

In recent decades, many efforts have been made to better balance the relationship of weight gain with feed intake in beef cattle; however, some traits are less effective to minimize the negative response of increased adult animal size, such as gross feeding efficiency [3,5]. In this respect, the residual feed intake (RFI) was proposed in the 1960s [6] and has gained more notability for being independent from growth and body size, and designed towards intake reduction [3,5-8]. This independence is attributed to the fact that RFI is calculated as the difference between observed and estimated intake by a regression equation of DMI over the average daily gain (ADG) and the mid body weight (MBW) [6].

Both DMI as RFI in cattle have enough variability and heritability to respond to genetic selection [2,7,8]; however, there is still no consensus on how these traits should be considered in the indices of selection. In this context, two major limitations comprise the difficulties inherent to obtain phenotypes and how to use this information in the breeding process [9]. In Nellore cattle (*Bos taurus indicus*), these difficulties are even greater, given the few animals with phenotype available for DMI and RFI, and the scarce knowledge about genetic parameters of these traits in Nellore. However, the phenotypic variability of RFI in Nellore show a standard deviation ranging between 0.31 and 0.69 kg/DM per day [10,11], is similar to that observed in *Bos taurus taurus* (often referred as taurine) animals and crossbreed [2,3,6,7].

The use of genomic information can be a strategy to improve the selection of phenotypes such as RFI and DMI, if the marker effects are estimated accurately. Genome-wide association study (GWAS) allowed to identify subsets of markers that explain an important portion of the variation of these characteristics [9,12,13]. The use of the information obtained from these markers along the chromosomes (BTA) can improve the accuracy of young animals candidates for genetic selection, and thus improve the genetic gain by reducing the generation interval.

Several studies have reported the viability of using the information from single nucleotide polymorphism (SNP) to identify regions of the genome that affect phenotypes of interest, aiming at improving breeding schemes for weight gain, reproduction and carcass traits in beef cattle [14-16]. Additionally, studies on molecular markers in cattle were enhanced with the recent release of the reference bovine genome [17] and with the improvement of beadchip technologies that perform fast and automated analyses of hundreds of thousands of SNPs and with the decreasing cost per SNP analyzed. The development of high-density commercial panels of SNPs opened a range of opportunities for GWAS [14]. Furthermore, the imputation of genotypes has proven to be an effective tool in enhancing the power of GWAS by increasing the number of genotyped animals and can be a valuable strategy for reducing even more the genotyping cost [18].

However, the vast majority of GWAS has been performed in animals of the taurine subspecies. Also, the first beadchip of thousands of SNPs were developed based on this subspecies, which causes several SNPs, described as being polymorphic in taurines, to be non-informative in zebu cattle (*Bos taurus indicus*), especially Nellore [19,20]. Only in recent years, GWAS was carried out more often in zebu from the development of optimized beadchips also taking into account this subspecies (e.g. [20]). The objectives of this study were: 1) to identify SNPs associated with DMI and RFI in Nellore cattle, using medium (Illumina® BovineSNP50 v2 BeadChip), high density (Illumina® BovineHD BeadChip) and a combined of medium to high-density panels by imputation; and 2) to explore the regions surrounding the identified markers seeking possible genes with known function near these SNPs.

## Results

### Phenotype, quality assurance and imputation

For the three datasets of the two phenotypic variables, no evidences were found to deviate from normality and homoscedasticity of model residuals in the Shapiro-Wilk and Breusch-Pagan tests (P > 0.05), respectively. The mean, additive genetic variances and residual variances were 8.76 ± 1.96, 0.29 and 0.42, respectively to DMI and 0.00 ± 0.89, 0.20 and 0.33, respectively to RFI. However, four samples were considered outliers in the DMI distribution and removed from the HD dataset and HDimp dataset, whereas two samples were considered outliers for RFI. Regarding the criteria for exclusion of samples and SNPs, the results of the quality control of 50 k and HD are shown in Table 1.

**Table 1:** Number and percentage of SNPs excluded in quality control

**Criterion**	**HD (SNPs)**	**HD (%)**	**50 k (SNPs)**	**50 k (%)**
Location*	42,669	5.5	1,723	3.2
Call rate	104,602	13.4	9,253	16.9
MAF	173,564	22.3	13,728	25.1
HWE	20,529	2.6	16,740	3.1
Total	341,374	43.9	26,378	48.3

*The criterion location excluded SNPs without coordinated genomic known and not located in autosomal chromosomes.

After the quality control criteria, the final datasets were: 672 samples in 50 k with 28,231 SNPs and 365 samples in HD with 436,588 SNPs.

The number of SNPs imputed with over 95% accuracy was 290,620 and the number of remaining samples was 672 for the dataset HDimp. The median imputation accuracy was 97.2% with an average of 94.6%. Only those genotypes imputed with over 95% accuracy were used in the GWAS.

### Genome-wide association study

**Figure 1:**
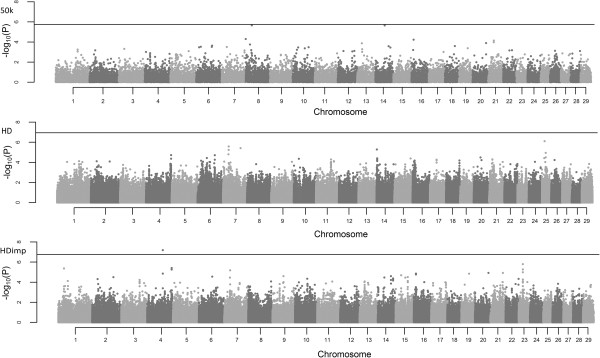
**Manhattan plots of –Log**_**10**_**(P-values) for DMI in Nellore cattle.** The horizontal lines represent the Bonferroni threshold (50 k = 1.77 × 10^-6^, HD = 1.15 × 10^-7^, HDimp = 1.72 × 10^-7^).

**Figure 2:**
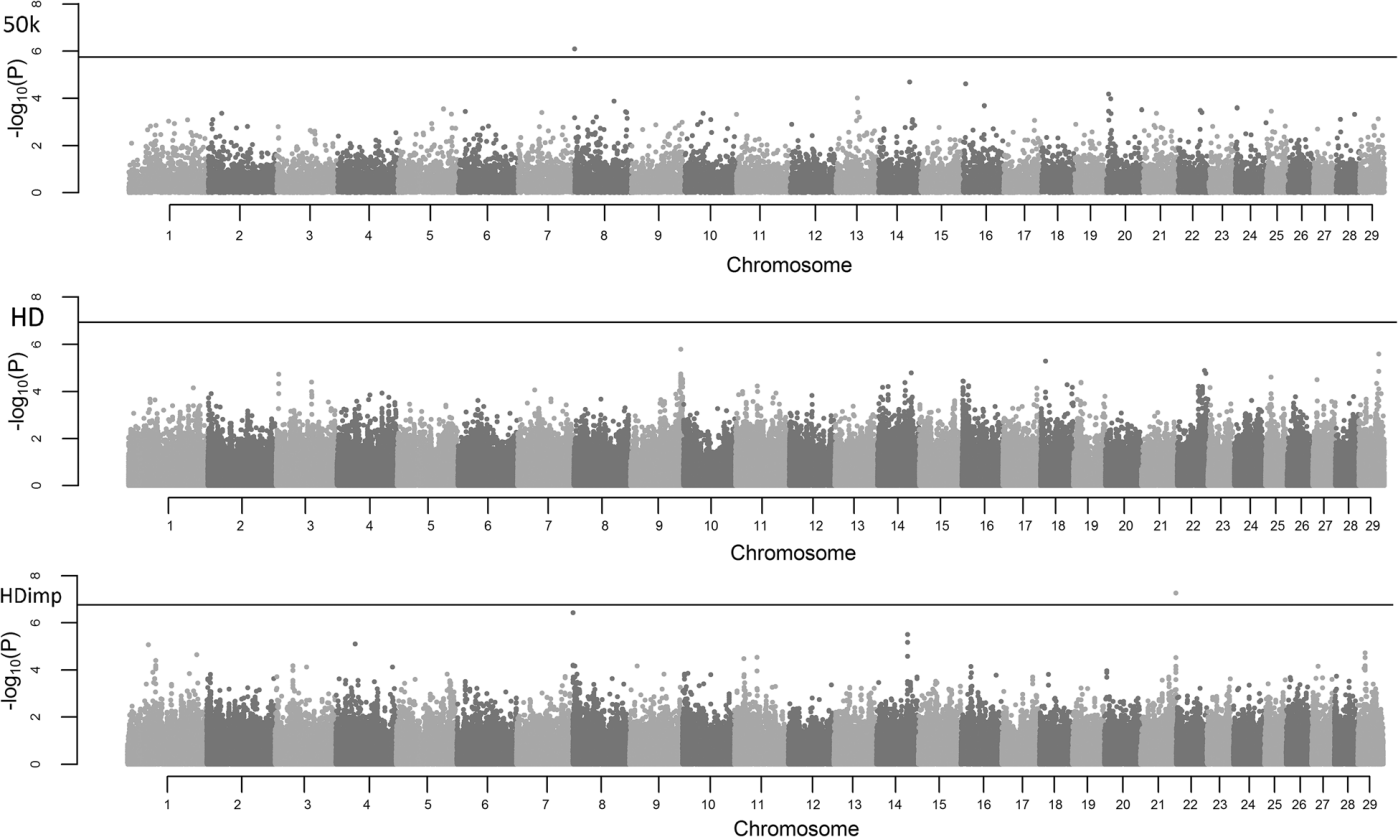
**Manhattan plots of –Log**_**10**_**(P-values) for RFI in Nellore cattle.** The horizontal lines represent the Bonferroni threshold (50 k = 1.77 × 10^-6^, HD = 1.15 × 10^-7^, HDimp = 1.72 × 10^-7^).

The deflation/inflation factor (λ) calculated for all association analyses was lower than 1.1 (Additional files 1, 2, 3, 4, 5 and 6), which was considered acceptable and can be used on genomic control correction approach (GC). The P-values of SNPs along the chromosomes are shown in the form of Manhattan Plots for DMI and RFI in Figures 1 and 2, respectively, with the threshold represented as the Bonferroni significance line.

The SNPs significant in the Bonferroni adjustment in each DMI and RFI analysis are described in Table 2.

**Table 2:** Description of the most significant SNPs for DMI and RFI

**Trait/dataset**	**SNP ID**	**BTA**	**BP (Mb)**^**1**^	**MAF**	**Ef. Sub.**^**2**^	**Var (%)**^**3**^	**P-value**
DMI/50 k	rs109784719	14	44.9	0,13	-1.83	9.59	2.28 × 10^-06^
DMI/50 k	rs29024524	8	28.7	0,10	1.60	7.64	2.22 × 10^-06^
DMI/HDimp	rs134003539	4	73.5	0.38	0.36	1.78	6.53 × 10^-08^
RFI/50 k	rs41660853	8	4.5	0.12	0.48	4.65	1.13 × 10^-07^
RFI/HDimp	rs135777172	21	71.0	0.09	0.89	11.1	5.37 × 10^-08^

^1^BP = position in base pairs.

^2^Ef. Sub = allelic substitution effect (DM/day).

^3^Var = proportion of the explained phenotypic variance.

The exploration of the region around the five SNPs that were associated with DMI and RFI are shown in Table 3, which shows all the genes with known functions located around (100 kb) these SNPs. Several other genes are at shorter distances; however, these distances were not included because their function is still unknown according to Ensembl genes 72 UMD v 3.1 [21]. Additionally, the distance of the QTLs mapped for both phenotypic variables closer to these SNPs is shown in the same table.

**Table 3:** Genes and QTLs that are close to SNPs associated with RFI and DMI

**SNP (trait)**	**Gene**	**Gene ID**^**a**^	**Dist gene (kb)**^**b**^	**Strand**	**Dist QTL (Mb)**^**c**^	**QTL ID**^**d**^	**Full gene name (description)**
rs109784719 (DMI)	*STMN2*	534991	27.4	+	44.1	#4365	Stathmin-like 2
rs29024524 (DMI)	*CCDC171*	538331	0	-	12.1	#4425	Coiled-coil domain containing 171
	*PSIP1*	282011	48.3	+			PC4 and SFRS1 interacting protein 1
	*SNAPC3*	511366	89.3	-			Small nuclear RNA activating comp 3
rs134003539 (DMI)	*ZNF804B*	100295505	0	+	4.5	#10584	Zinc finger protein 804B
rs41660853 (RFI)	*ANXA10*	505322	0	+	7.1	#4353	Annexin A10
	*DDX60*	787280	0	-	7.9	#5274	DEAD (Asp-Glu-Ala-Asp) box polypep 60
rs135777172 (RFI)	*GPR132*	539146	14.8	+	2.2	#4462	G protein-coupled receptor 132
	*CDCA4*	527837	40.7	-			Cell division cycle associated 4
	*AHNAK2*	527701	84.9	-			AHNAK nucleoprotein 2
	*BRF1*	618161	93.3	-			RNA polymerase III transcr init factor 90

^a^Identification of the gene according to Ensembl genes database 72.

^b^Distance in kb of SNP for the gene.

^c^Distance in Mb of SNP for the closer QTL described in trait associated with SNP.

^d^Identification of QTL according to the cattle QTLdb database.

## Discussion

The thresholds for the SNP exclusion are still questionable. The accuracy load of each criterion depends heavily on the dataset and information that it is expected from each type of GWAS. The criterion that eliminates most SNPs and, possibly, the most controversial is MAF. In general, there is a tendency to use 5% for GWAS [9,14,16,22-24]; however, in this study, we adopted 2% of MAF, even with approximately 25% of SNPs being eliminated in this criterion. Other studies also adopted the same level [20,25,26].

The GWAS across the 29 autosomal chromosomes showed no genomic region associated with DMI and RFI of feedlot Nellore young bulls and steers, as expected for the variables that have their regulation controlled by many genes. This fact was reported in other studies that also assessed DMI and RFI in different cattle subspecies [9,14] and RFI in pigs [27].

The benefits of using imputation were observed in this study because, in the dataset HDimp, we found regions significantly associated with phenotypes, which was not noted in the dataset HD even though both had a very close Bonferroni threshold. Although the number of SNPs of the dataset of imputed animals is much lower than that of the dataset HD (290,620 *vs* 436,588), this tool enabled a much more robust association study due to the considerable increase in the number of samples (672 *vs* 365).

The two variables showed high genetic and phenotypic correlation among each other [2,8,28], but not always the same regions showed strong association with these characteristics. This can be partly explained by the difference between the physiological mechanisms that regulate RFI are not exactly the same that regulate DMI. On the other hand, regions with important effect on the two traits suggest the existence of pleiotropic effects on these variables [29,30]. However, some regions are well evidenced in both analysis of the same trait and, in some cases, we can observe genomic regions that relate to both, such as in BTA4, BTA8 and BTA14.

Three SNPs surpassed the threshold for the Bonferroni multiple test for DMI and two SNPs for RFI. Several markers have been associated with these two variables in the literature [9,12,14,22-24,27,29,30]; however, the methodologies used for this purpose are diverse and populations assessed are extremely distinct, which may imply that associations made in a particular breed may not be applied in others [31]. These SNPs can explain part of the phenotypic variance, insomuch that few markers explain more than 30% of the variation in RFI [9,27,30]. However, this calculation takes into account allele frequencies, the allele substitution effect and phenotypic variance of the trait. This prediction can be overrated depending on these factors, mainly when it assumes independence between the markers considered in this calculation. The allele substitution effect of the SNPs varied between the panels, and this effect in DMI was higher for markers in the 50 k panel, for RFI in the HDimp panel.

Regarding the location of SNPs related to DMI, the SNP rs109784719 (BTA14) is at 27.4 kb of the single gene (*STMN2*); however, it is in the region of QTL #4365 described previously for DMI. Other studies found SNP associated with DMI in the BTA14 in beef cattle [9,24] and in chromosome 14 in pigs [32], both in a region surrounding the genes such as *PLAG1*, *RDHE2* and *CHCHD7* that notably influence the stature of various species [20,24,33]. The SNP rs29024524, in the BTA8, is in gene *CCDC171* and surrounding other genes; moreover, it lies next to the QTL #4425, which is a genomic region that seems to affect RFI and DMI.

Other two QTLs (#4353 and #5274) involved with RFI surround this SNP and SNP rs41660853, associated with RFI. This SNP linked to RFI is located near gene *CLCN3*, related to the ionic transport processes already reported as influential in RFI [12], corroborating a previous study that described the metabolic differences of RFI [34]. Three other association studies indicated the importance of this region in RFI and DMI [14,23,29].

The ionic transport system can account for more than 10% of all ruminant energy expenditure [35]; therefore, for animals with lower energy expenditure to maintain this system, they can, at the same time, direct the energy consumed to other processes, which can influence feeding efficiency. The SNP rs135777172 also lies in a region of QTL (#4462) already described for RFI at final part of BTA21, where there is an abundance of genes encoding various types of proteins [21].

Finally, SNP rs134003539 is in a locus described as important, for both DMI and RFI [12,14,23,29,36,37], at 4 Mb of the QTL (#10584). Other two genes widely studied in both phenotypic variables are also located in this locus. The neuropeptide Y (*NPY*, Gene ID 504216) positioned at 1.4 Mb of distance and the leptin (*LEP*, Gene ID 280836) at 19 Mb from the SNP and inserted in this QTL. The neuropeptides and hormones that control appetite, energy expenditure and metabolism of fat and glucose have a relationship to each other, which seems to be mediated by leptin [38]. These compounds can increase appetite such as the neuropeptide Y, ghrelin, *AGRP, MCH*, orexines and noradrenalines, or they can even have a reducing effect on appetite such as leptin, *POMC, CART, CRH, α-MSH* and serotonin [38]. In addition, the rs134003539 is in gene *ZNF804B* that is a form of protein “zinc finger”, characterized by coordination and stabilization of one or more zinc ions in several ionic exchange processes [39].

In general, the GWAS presents itself as an interesting tool to identify genomic regions that can influence these phenotypes. Another potential advantage is the creation of less dense panels designed for a breed or subspecies of interest containing SNPs directed to a certain characteristic. This could reduce costs of a possible genetic selection with the use of genomic data, improving the accuracy of estimates of genetic value in animals.

The identification of regions associated with DMI and RFI may elucidate loci that influence these variables and highlight possible important physiological mechanisms. In this study, some promising regions were identified, with important effect on DMI and RFI. However, the strict significance level adopted and the number of genotyped animals may have contributed to reducing the power of the present study, given that important regions may not have been identified, which suggests the need for further studies aimed at overcoming such restrictions.

## Conclusions

The current study showed the results of GWAS in high and medium-density panels to identify genomic regions associated with feed intake and efficiency. It is believed to be the first study of this kind conducted in Nellore cattle to these traits. The results suggest that RFI and DMI are influenced by loci previously described and these results obtained in zebu cattle are supported by the literature.

## Methods

### Phenotypic records

The study was conducted from a compilation of 11 experiments of feed efficiency and intake conducted in Brazil from 2007 to 2011. One experiment was performed in the South [28], eight in the Southeast [10,19] and two in the Midwest region in Brazil. In these experiments, we obtained phenotypes of 720 young bulls and steers at 550 ± 115 days old, with body weight of 380 ± 51 kg. The sample size of each trial ranged from 50 to 120 animals. Three different facilities were used to measure the phenotypes. We used automated systems of collective stalls (Calan gates and GrowSafe™) and an individual pens system. The experiment lasted at least 70 days, and the dietary intake was measured daily with subsequent adjustment for dry matter content.

During the experiments, the MBW of each animal was obtained by periodic weighing without fasting and ADG was calculated as the slope of the linear regression of weights compared to the testing days. To calculate the RFI, we considered the regression equation residue of DMI on ADG and MBW [6]; however, because of the different experiments, we formed contemporary groups (CG), which means that animals of the same experiment were considered as a CG. Within three of the CG, there were young bulls and castrated steers; therefore, the sexual condition (SC) was also included as a covariate in the statistical model. The RFI was calculated in the PROC REG procedure from the statistical package SAS 9.3 and the general equation was:

$$\mathrm{DMI}=\beta_{0}+\beta_{1}\mathrm{ADG}+\beta_{2}\mathrm{MB}W^{0,75}+\beta_{3}\mathrm{SC}+\beta_{4}\mathrm{CG}+\epsilon$$

RFI and DMI were tested for data normality (Shapiro-Wilk) and homoscedasticity of model residues (Breusch-Pagan). We also performed the control of data outliers (DMI, ADG, MBW and RFI), in which the records outside the mean range ± 3 standard deviations, classified as possible measurement error, were excluded from the analysis.

### DNA extraction, sample assurance and SNP quality control

Blood was collected from all animals evaluated by puncturing the jugular. We used tubes containing K2 EDTA as anticoagulant. The samples were stored at 4°C for late DNA extraction by NaCl precipitation [40]. After extraction, the quality of the DNA samples was assessed by determining the ratio A260/280 in biophotometer. The samples were only accepted when values remained between 1.8 and 2.0 concomitantly, the DNA was, then, quantified and samples were diluted to a minimum concentration of 50 ng/μL and maximum of 150 ng/μl for subsequent genotyping.

Genotyping was performed in two types of DNA beadchip: the Illumina® BovineHD BeadChip (777,962 SNPs) [41], and the Illumina® BovineSNP50 v2 BeadChip (54,609 SNPs) [42], both in the standard test Infinium Assay II for the HiScanSQ® platform (Illumina Inc., San Diego, CA). Genotype calls were determined using the validated standard cluster file provided by the manufacturer, with GenCall Scores greater than 0.70. In total, 720 animals were genotyped, including 336 young bulls and steers in BovineSNP50 (50 k dataset) and 384 young bulls in BovineHD (HD dataset). As most of the BovineSNP50 markers are embedded in the BovineHD panel, the HD samples were also included in the 50 k dataset in order to increase sample size. We assessed the occurrence of duplicate samples by calculating the proportion of alleles identical by state (IBS) between all possible pairs of individuals. For IBS calculation, genotypes were considered for 5,000 and 20,000 markers taken randomly for BovineSNP50 and BovineHD, respectively, and any pair of samples with IBS ≥ 95% were excluded.

For GWAS, SNPs were subjected to a quality control in which only autosomal SNPs with known genomic coordinate were considered. Samples with Call rate (IDCR) lower than 90% were removed from the study. Markers were removed if they presented minor allele frequency (MAF) ≤ 0.02, Call rate (SNPCR) ≤ 0.95 and P-value for Fisher’s exact test for Hardy-Weinberg Equilibrium (HWE) ≤ 1 x 10^-5^. This quality control was performed in R v2.15.2 software using scripts developed for this purpose [20] and the GenABEL v1.7-6 package [43].

### Imputation

In order to increase sample size, an imputation analysis was performed to combine the available information of animals genotyped with BovineSNP50 and BovineHD. The HD panel information was used to verify the imputation efficiency from the 50 k Panel to the HD. The quality control of SNPs was performed again, which excluded SNPs located in non-autosomal chromosomes, SNPCR ≤ 0.97, HWE ≤ 1 × 10^-6^, IDCR ≤ 0.90.

Afterwards, 290 animals randomly sampled (genotyped in high-density panel) were considered as population reference for the imputation analysis, while the remaining animals of the panel were included in a validation set. Except for the markers in common between the two technologies, the genotyped animals in the validation BovineHD had their genotypes masked and imputed, which allowed to simulate the imputation of 50 k to HD. These analyses were performed using the FImpute 2.2 software [44], using HD genotypes in combination with pedigree information. The imputation efficiency was assessed using the proportion of genotypes imputed correctly. Subsequently, imputation was performed similarly to all animals in the 50 k, in which all genotyped animals in HD that passed the quality control criteria (362) were considered as reference population. The final dataset (HDimp dataset) was composed of 672 animals and 290,620 SNPs that were allocated with accuracy greater than 95%.

### Association analysis

The association analysis was based on a variance-components method, namely Grammar-Gamma [45]. This method is a computationally efficient unbiased approximation of the gold standard likelihood ratio test (LRT) [45,46], which corrects the association analysis for confounding due to genetic substructure and relatedness.

The first step in the association analysis was to use the variance-covariance matrix as genomic relationship matrix, to correct for relatedness and substructure and Grammar-Gamma factor calculation. To ensure the reliability of the estimates, we calculated the inflation/deflation factor (λ) for the correction of the GC [47].

The second step consisted of associating the phenotype with the genotypes without the variance-covariance matrix and the estimates of SNPs effects adjusted by the Grammar-Gamma factor [47]. The model used was the polygenic [48] and the association test varied depending on the phenotype used. The polygenic model for RFI included age and SC as covariate and, for DMI was included SC and MBW as covariates. The results were presented as Manhattan Plots in which the -log_10_(P- values) were plotted, corrected for λ and the Bonferroni correction (α = 0.05/number of SNPs) was considered as significance threshold. All the procedures described in this item were carried out in R v2.15.2, using the GenABEL v1.7-6 package [43].

### Region surrounding significant SNPs

The SNPs that surpassed the threshold of the Bonferroni adjustment were described and their allelic substitution effects were reported. The proportion of the phenotypic variance explained by the SNPs was also estimated as:

$$\mathrm{VAR}\left(\%\right)=\frac{2\mathrm{pq}\beta^{2}}{S^{2}}*100$$

Where p and q are the allele frequencies of the analyzed SNP, β^2^ is the square of the allele substitution effect and S^2^ is the total variance of phenotype.

Additionally, they were investigated for their genomic location (genes surrounding and possible QTLs already mapped for DMI and RFI). The exploration of the region searched for genes with known functions located at the maximum 100 kb distant from the SNPs in Ensembl genes 72 using the assembly UMD v3.1 [21]. The search for QTLs was examined in cattle QTLdb database [49].

## Authors’ contributions

JBSF coordinated and designed the study. MHAS, YTU, RCG, JFG, HF, SLL, PRL, TGL and JBSF contributed in study design and obtaining phenotypes. YTU, MHAS and HHRN helped in data analysis and imputation. MHAS, PAA, RCG and HF did the extraction and standardization of DNA samples from the animals. LLC and RAB performed the genotyping of animals. MHAS performed data analyzes, phenotype collection and preparation of the manuscript. JBSF, YTU, MHAS, RCG, GAOJ, JFG FVM HF and interpreted results and edited the manuscript. All authors approved the final version of the manuscript.

## Supplementary Material

Additional file 1 Quantile-quantile plot for the test statistics used in the association analysis for DMI (50K). Click here for file

Additional file 2 Quantile-quantile plot for the test statistics used in the association analysis for DMI (HD). Click here for file

Additional file 3 Quantile-quantile plot for the test statistics used in the association analysis for DMI (HDimp). Click here for file

Additional file 4 Quantile-quantile plot for the test statistics used in the association analysis for RFI (50K). Click here for file

Additional file 5 Quantile-quantile plot for the test statistics used in the association analysis for RFI (HD). Click here for file

Additional file 6 Quantile-quantile plot for the test statistics used in the association analysis for RFI (HDimp). Click here for file
