# Supplementary figures and images for: Genome-wide association analysis of feed intake and residual feed intake in Nellore cattle
Source: BMC Genet. 2014 Feb 11;15:21. doi: 10.1186/1471-2156-15-21 (PMC3925773; doi:10.1186/1471-2156-15-21)

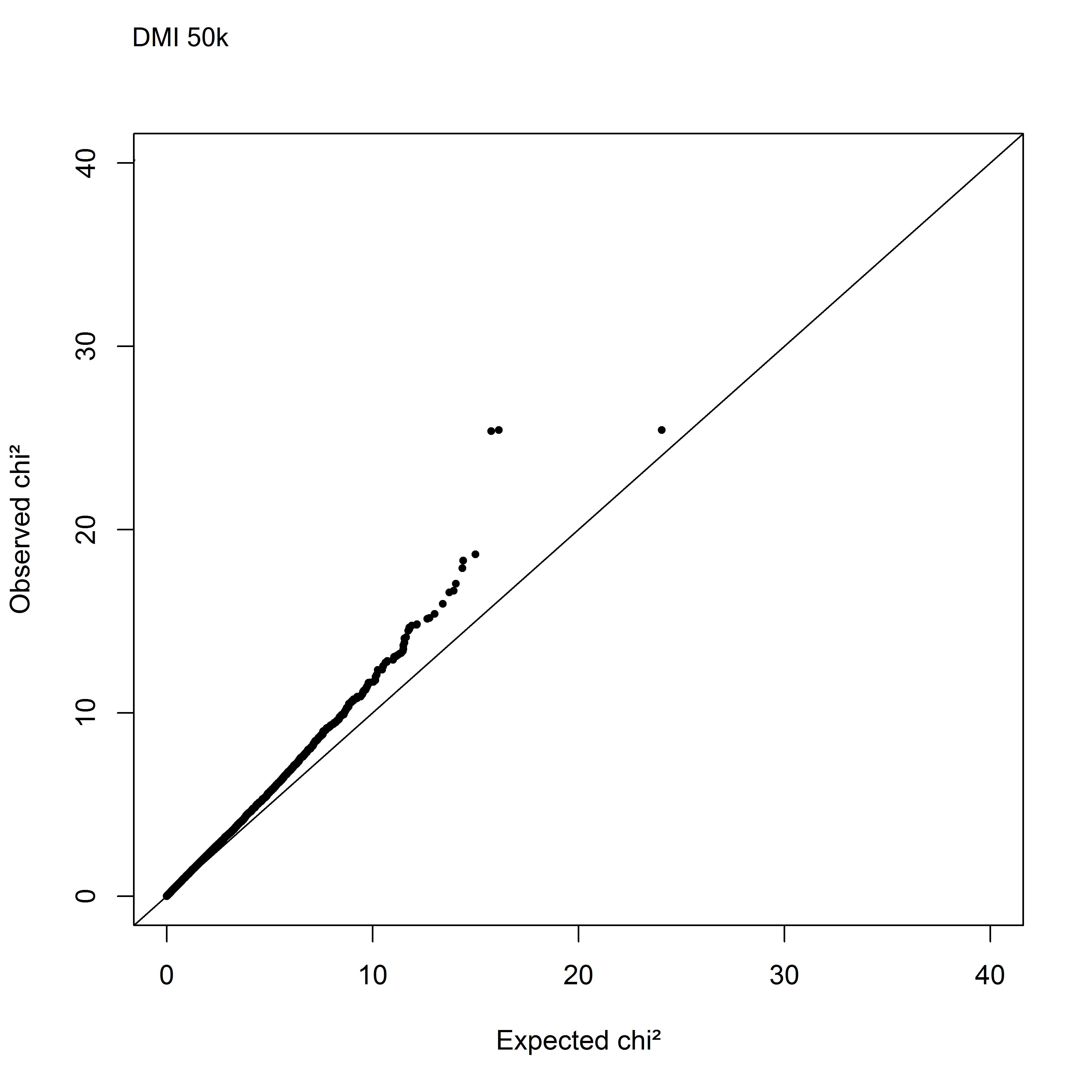

Supplement: Additional file 1 — Quantile-quantile plot for the test statistics used in the association analysis for DMI (50K). [file 1471-2156-15-21-S1.tiff]

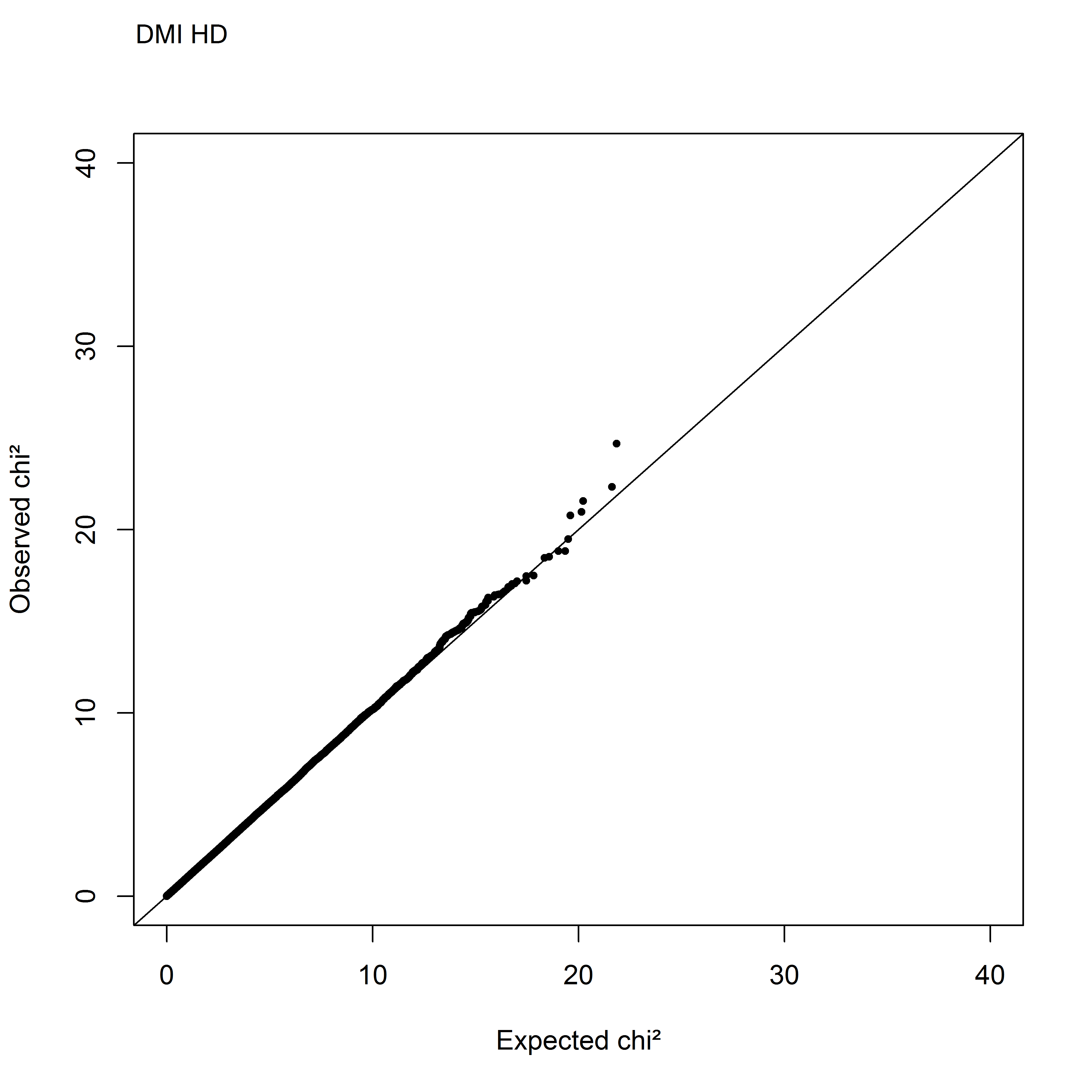

Supplement: Additional file 2 — Quantile-quantile plot for the test statistics used in the association analysis for DMI (HD). [file 1471-2156-15-21-S2.tiff]

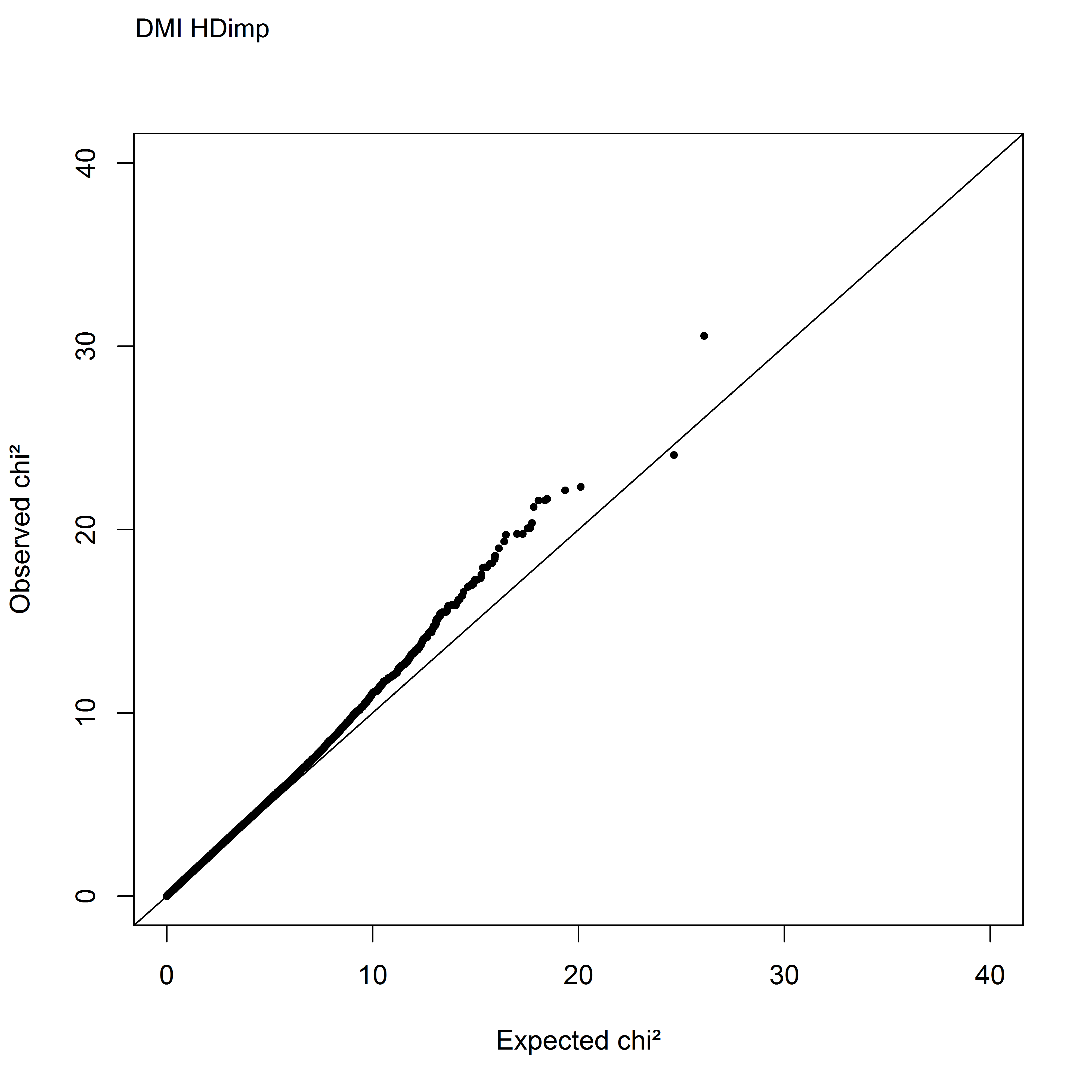

Supplement: Additional file 3 — Quantile-quantile plot for the test statistics used in the association analysis for DMI (HDimp). [file 1471-2156-15-21-S3.tiff]

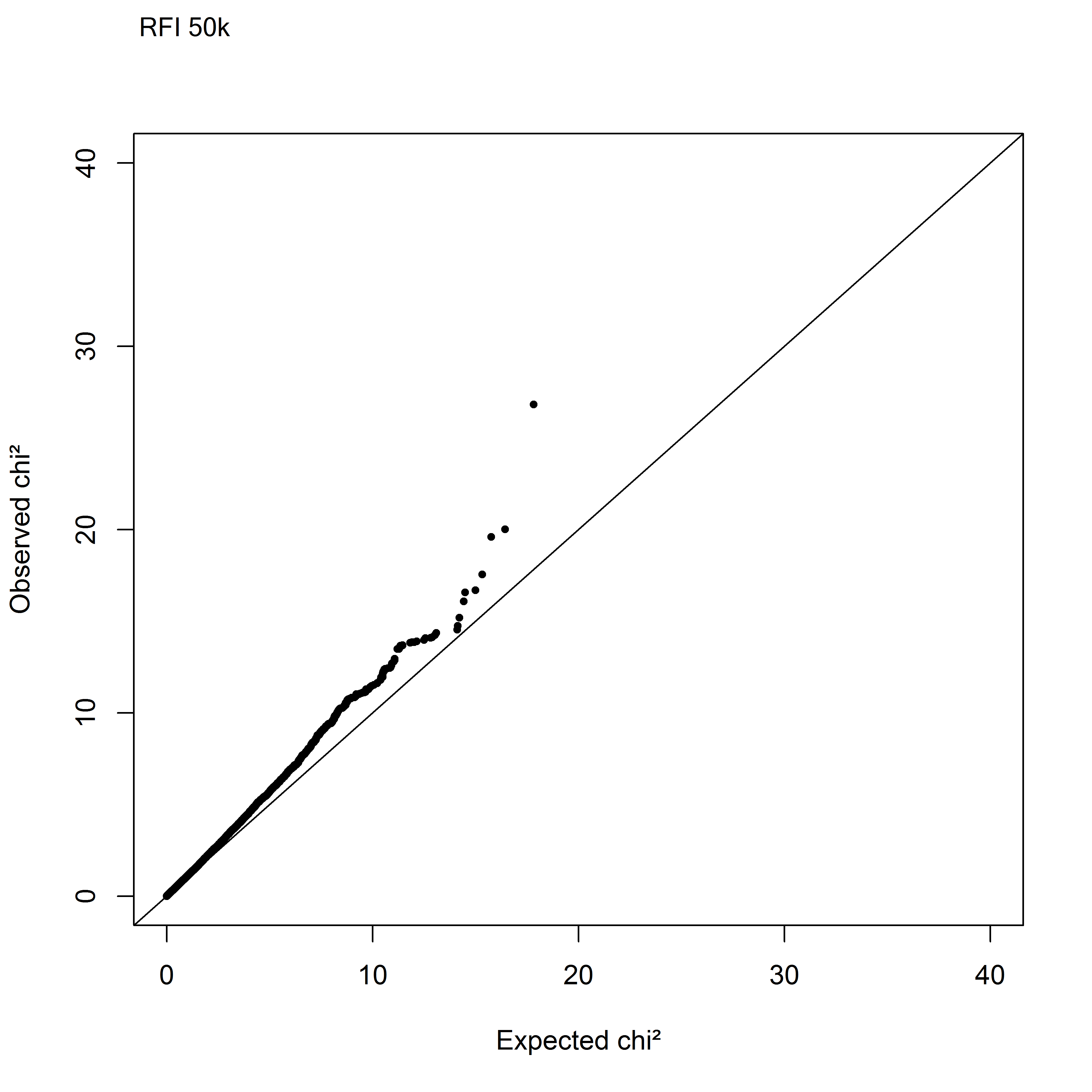

Supplement: Additional file 4 — Quantile-quantile plot for the test statistics used in the association analysis for RFI (50K). [file 1471-2156-15-21-S4.tiff]

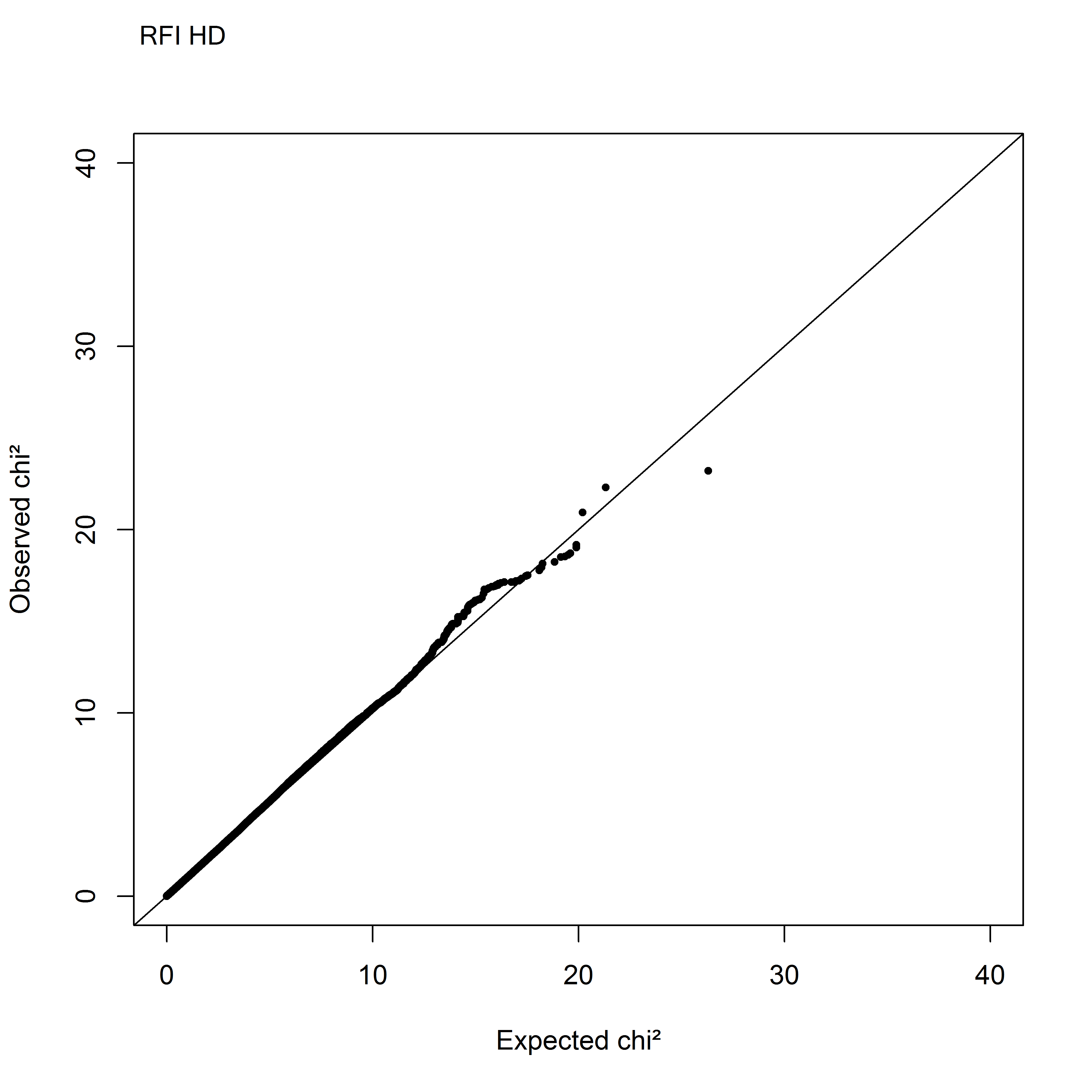

Supplement: Additional file 5 — Quantile-quantile plot for the test statistics used in the association analysis for RFI (HD). [file 1471-2156-15-21-S5.tiff]

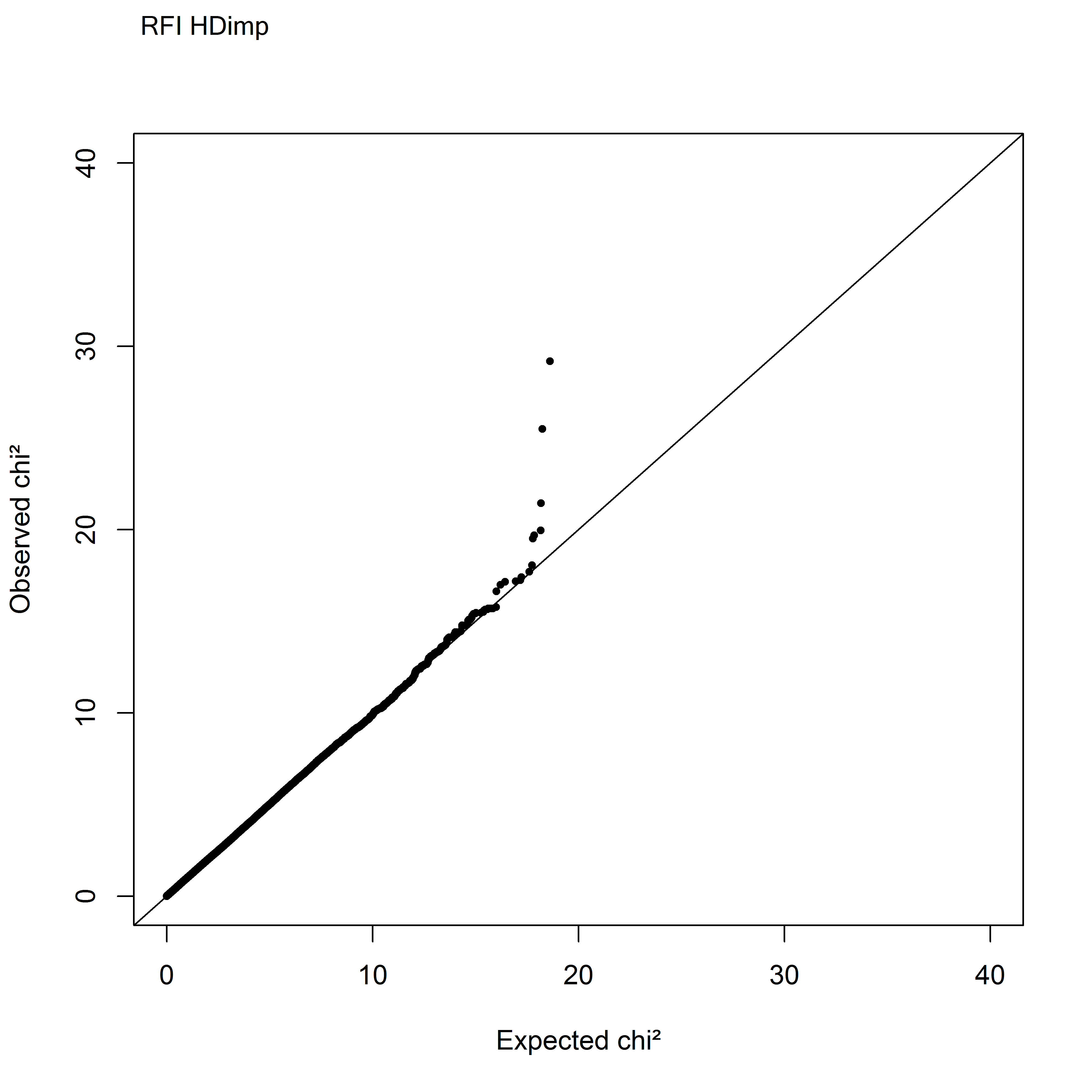

Supplement: Additional file 6 — Quantile-quantile plot for the test statistics used in the association analysis for RFI (HDimp). [file 1471-2156-15-21-S6.tiff]
